# The correlation between colorectal cancer rates of proliferation and apoptosis and systemic cytokine levels; plus their influence upon survival

**DOI:** 10.1038/sj.bjc.6603104

**Published:** 2006-04-25

**Authors:** C Evans, I Morrison, A G Heriot, J B Bartlett, C Finlayson, A G Dalgleish, D Kumar

**Affiliations:** 1 Colorectal Surgery Unit & Division of Oncology, St James Wing (Level III), St George's Hospital, Blackshaw Road, London SW17 0QT, UK

**Keywords:** colorectal cancer, cytokines, immune suppression, proliferation: apoptosis, survival

## Abstract

Colorectal cancer development is associated with a shift in host immunity with suppression of the cell-mediated immune system (CMI) and a predominance of humoral immunity (HI). Tumour progression is also associated with increased rates of cell proliferation and apoptosis. The aim of this study was to investigate whether these factors correlate and have an influence upon prognosis. Long-term follow-up was performed on 40 patients with colorectal cancer who had levels of tumour necrosis factor (TNF)-*α*, interferon (IFN)-*γ* and interleukin (IL)-10 measured from stimulated blood cultures before surgery. Their archived tumour specimens were analysed to determine a Ki-67-derived proliferation index (PI) and a M30-derived apoptosis index (AI). Tumour necrosis factor-*α* levels negatively correlated to tumour proliferation (*ρ*=−0.697, *P*=0.01). Interleukin-10 levels had a positive correlation with tumour proliferation (*ρ*=0.452, *P*=0.05) and apoptosis (*ρ*=0.587, *P*=0.01). Patient survival correlates to tumour pathological stage (*P*=0.0038) and vascular invasion (*P*=0.0014). An AI⩽0.6% and TNF-*α* levels ⩾8148 pg ml^−1^ correlate to improved survival (*P*=0.032, *P*=0.021). Tumour proliferation and apoptosis correlate to progressive suppression of the CMI-associated cytokine TNF-*α* and to and higher levels of IL-10. Survival is dependent upon the histological stage of the tumour, vascular invasion, rates of apoptosis and proliferation and systemic immunity which are all interconnected.

The basis for the development of colorectal cancer is believed to be the adenoma-carcinoma sequence (Weitz *et al*, 2005), which involves a stepwise progression from normal to dysplastic epithelium to carcinoma. It is associated with the accumulation of multiple, clonally selected genetic alterations (Leslie *et al*, 2002) and results in disturbed rates of cell proliferation and apoptosis (Aotake *et al*, 1999).

Cell proliferation and apoptosis are dependent upon the pathways that govern the different cell cycle phases. These pathways are under genetic control but are influenced by both exogenous and endogenous stimuli (Funk, 1999). The immune system and its components are key regulators of cell proliferation and cell death when there are inflammatory responses, for example, during wound healing (Robinson and Coussens, 2005). The majority of colorectal cancer evolves in a chronic inflammatory background (Higaki *et al*, 1999) and there is strong evidence of an immune response to colorectal cancer (Naito *et al*, 1998; Garrido and Algarra, 2001; Nagorsen *et al*, 2005).

In order for tumours to establish and progress it is thought that suppression of the host's immune system is needed (Berghella *et al*, 1996). Tumour-induced immune suppression occurs at both a molecular and cellular level and progresses to affect the whole organism (Zou, 2005). Colorectal cancer results in a fundamental shift in immunity with suppression of the cell-mediated immune system (CMI), associated with tumour rejection (Kidd, 2003), and a predominance of humoral immunity (HI) which conversely prevents rejection (Terabe *et al*, 2004). There is a reduced production of cytokines from the CMI-associated Th1 lymphocytes while those produced by the Th2 lymphocytes (associated with HI) appear to remain at normal or even elevated levels. The further the disease has progressed the more significant these imbalances become and complete resection of the tumour reverses these changes (O’Hara *et al*, 1998; Heriot *et al*, 2000; Galizia *et al*, 2002; Shibata *et al*, 2002).

The purpose of this study was to correlate colorectal tumour rates of proliferation and apoptosis with host systemic cytokine levels and investigate what influence these factors have upon patient prognosis. Archived cancer specimens from a group of patients, who were previously studied to investigate the relationship between systemic cytokine levels and colorectal cancer (Heriot *et al*, 2000), were stained with the antibody Ki67 to measure proliferation and the antibody M30 to measure apoptosis. Long-term patient follow-up was also preformed. The original work had shown a suppression of CMI-associated cytokines, tumour necrosis factor-*α* (TNF-*α*) and interferon-*γ* (IFN-*γ*) in patients with colorectal cancer that was greater in those with a Dukes’ C tumour compared to those with either a Dukes’ A or B tumour and was reversed by surgery. No difference had been found in the levels of the HI-associated cytokine interleukin-10 (IL-10) in patients with colorectal tumours compared to the controls both pre- and postsurgery (Heriot *et al*, 2000).

## PATIENTS AND METHODS

Between March 1997 and March 1998, 53 patients (34 male patients) diagnosed at one institution with primary colorectal cancer were recruited. All patients gave written, informed consent to the study. Ethical approval for the study was obtained from the local ethical committee.

All patients had adenocarcinoma of the colon and rectum. Before treatment a single heparinised venous blood sample was taken from each patient for analysis. Following surgery a further venous samples was taken a minimum of 4 weeks postsurgery to be used for postoperative data analysis. The blood was diluted with RPMI-1640 medium (1 : 4) + glutamine (2 mM) and then stimulated for 24 h with lipopolysaccharide. Cell-free supernatants were then assayed for TNF-*α*, IFN-*γ* and IL-10 by enzyme-linked immunosorbent assay (ELISA) using an assay procedure and reagents (anticytokine capture monoclonal antibody, biotinylated anticytokine detecting antibody and recombinant cytokine) provided by Pharmingen (Cambridge Bioscience, UK) (Heriot *et al*, 2000).

Clinical follow-up was performed every 3 months for the first 2 years after treatment, 6 monthly during the third year and annually thereafter till the age of 80 years. In December 2004 patients with preoperative cytokine levels were followed up and their archived pathological specimens were reviewed retrospectively. Patient's who had chemotherapy and/or radiotherapy before surgery, patients with synchronous, or metachronous tumours, inflammatory conditions or any immunosuppressive condition including immunosuppressive medications were excluded. The interval from the date of resection to the date of death or last contact (if alive) was used for survival analysis with a minimum length of follow-up set at 5 years. The end points of the follow-up study were; cancer-related survival and the development of local recurrence or distant metastases.

### Immunohistochemistry

Sections (3 *ì*m thick) were cut from paraffin blocks on to SuperFrost® Plus slides (Menzel-Glaser, Germany), dried overnight at 60°C, dewaxed and taken down to water. Endogenous peroxidase activity was blocked by placing slides in 500 ml of a 10% hydrogen peroxidase solution for 8 min. Slides were washed in tap water and heated for 8 min in 1000 ml of Tris-EDTA-citrate buffer, pH 7.8 (preheated for 24 min) in a microwaveable pressure cooker if for Ki67 staining and for 35 min in 1000 ml of Tris-EDTA pH 9.9 in an unpressurised container if for M30 staining. After repeat washing, the slides were ringed with a hydrophobic ImmEdge™ pen (Vector Laboratories Inc., Bulingame, CA 94010, USA) and rinsed in Optimax Wash Buffer® (Biogenex Laboratories, San Ramon, CA, USA). The primary antibodies NCL-Ki67p (Novocastra Laboratories Ltd, Newcastle upon Tyne, England, UK) and M30 CytoDEATH (Roche Applied Science, Lewes, England, UK) were applied to the sections according to the Optimax stainer programme having been diluted to 1 : 6000 and 1 : 50, respectively, with ChemMate™ Antibody Diluent (DakoCytomation). The stainer was programmed using Optimax™ Software Version 2.7 to the following specifications: (1) incubation in primary antibody, 30 and 45 min, respectively, (2) 30 or 45 min, respectively, in polymetric conjugate, (3) 10 min in working substrate solution; rinsing between all stages with Optimax wash buffer®. Slides stained with M30 had the working substrate solution (ChemMate™ Envision™ Detection kit, Peroxidase/DAB, Rabbit/mouse (DakoCytomation) applied 45 min after the polymetric conjugate. On completion of the staining, the slides were washed in tap water, and counterstained with Mayer's haematoxylin for 1 min and then differentiated briefly in 1% acid alcohol, blued in running tap water, dehydrated cleared and mounted.

### Evaluation of staining results

**Figure 1:**
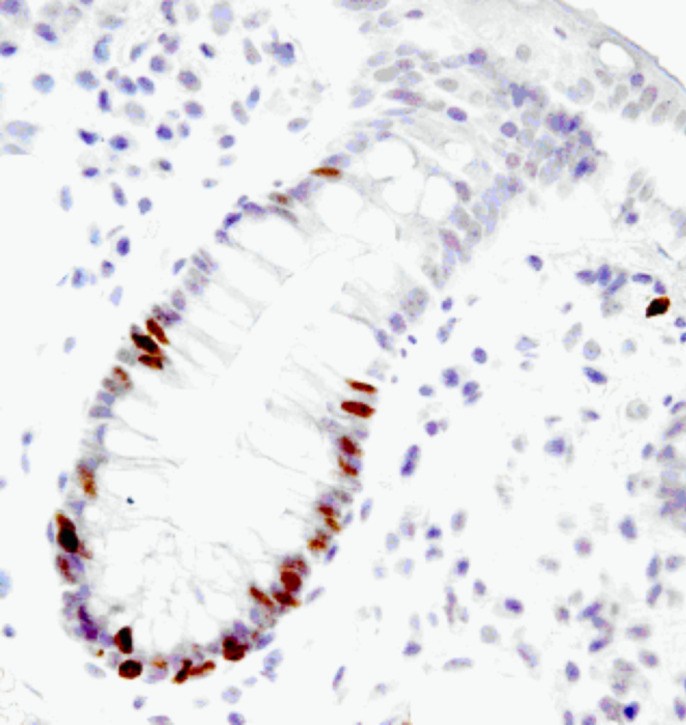
Histological section stained with anit-Ki67, 1 : 6000. Pretreatment Tris-EDTA pH7.6 (magnification × 200).

**Figure 2:**
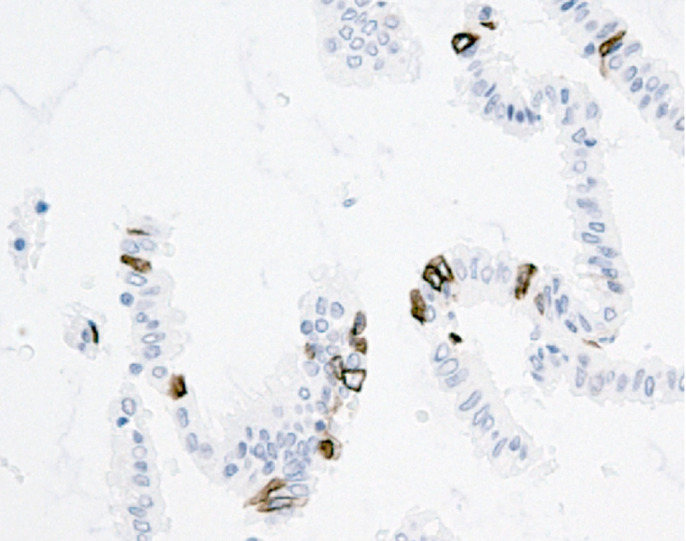
Histological section of colon stained with M30CytoDEATH 1: 50 TE buffer in the Decloaker (magnification × 200).

All specimens were examined by an independent investigator with no knowledge of the clinical or pathological data. Counts were made randomly from each slide with at least 1000 epithelial cells counted in all cases. Ki67 positivity and M30 positivity were identified and positive cells were expressed as a percentage of the total number of cells counted (proliferation index (PI) and apoptotic index (AI)) (Figures 1 and 2).

### Statistical analysis

Results are noted as mean +/− standard deviation (s.d.). Correlations between proliferation and apoptotic activity and systemic cytokine levels were assessed using the Spearman test. Independent *t*-tests were used to analyse means. In order to perform survival analysis and create probability curves dependent upon tumour rates of proliferation and apoptosis as well as systemic cytokine levels the Kaplan–Meier method was used. A log-rank test was used to compare the probability curves. Hazard ratios and 95% confidence intervals (CI) for cytokine values, PI, AI, Dukes’ stage, differentiation and vascular invasion were calculated for each factor in a Cox proportional hazards model. In all cases, a *P*-value of < 0.05 indicated a significant difference. All data were analysed using SPSS® Version 12.0 (SPSS Inc., Chicago, IL, USA).

## RESULTS

### Patient characteristics

From the original 53 patients, 40 (26 male patients) were eligible for the study. Of them, 11 were excluded for having no preoperative cytokine values successfully measured, one for the tumour being a local recurrence and one for being lost to follow-up. Median age was 76.5 years old with a mean (s.d.) of 74.55 (11.80). In all, 35 were Caucasian, four were Asian and one was Afro Caribbean. From this group of 40 patients, it was possible to retrieve and successfully stain 31 histological specimens for proliferation and apoptosis.

In December 2004, the median follow-up was 89 months (mean (s.d.) 87.64 (4.02) months). Of the patients, four had Dukes’ A tumours, 16 Dukes’ B tumours and the remaining 20 had Dukes’ C tumours. In all, 24 patients were documented to have well or moderately differentiated tumours while 15 had poorly differentiated tumours. Vascular invasion was found in 14 patient's specimens. Overall mortality was 65% (26 patients) with a cancer-related mortality of 50% (20 patients). Median survival was 21.5 months with a mean (s.d.) of 35.88 (33.64) months. Six patients at the time of surgery had metastases with an additional five developing metastases during follow-up. Five patients developed local recurrence postsurgery. Cancer-related 5-year mortality in this cohort of patients is noted to be high but was comparable to national statistics (Cancer Stats: Large Bowel-UK, Cancer Research UK, 2005) given the small number of patients involved.

### Proliferative and apoptosis activity

The mean (s.d.) PI in the colorectal carcinomas was 52.39 (19.50) %. The mean (s.d.) AI was 0.74 (0.6) %. Proliferative index and AI did not correlate to Dukes’ histological stage but, as a combined group, those patients who developed either local recurrence or metastases had a significantly raised PI (*P*=0.002) and AI (*P*=0.037). There was a strong positive correlation between AI and PI (*ρ*=0.675, *P*=0.01).

### Correlation between systemic cytokine levels and tumour rates of proliferation and apoptosis

**Figure 3:**
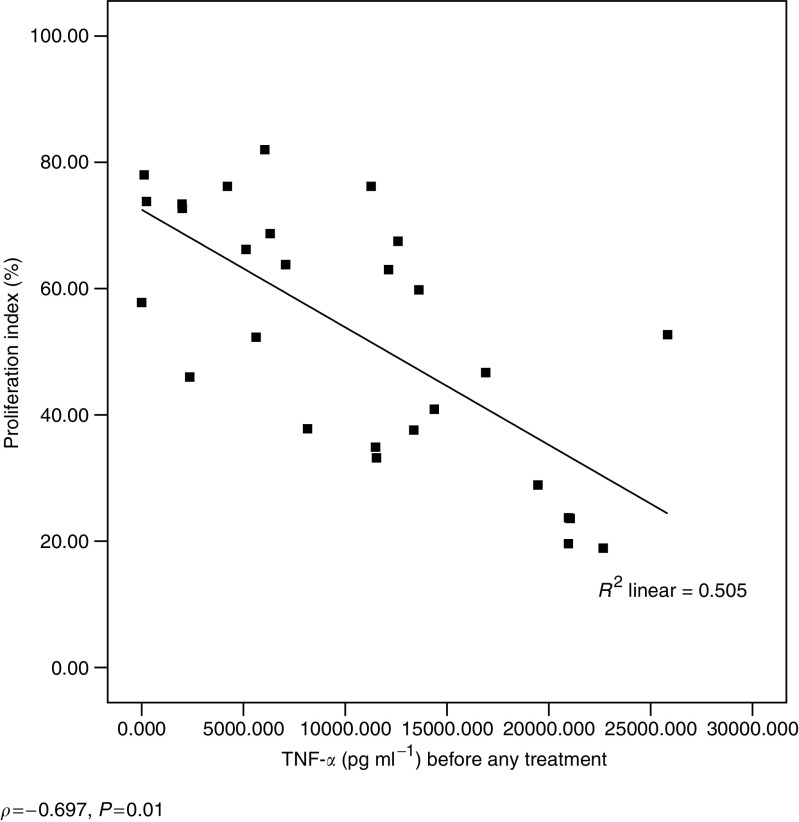
Correlation between preoperative TNF-*α* levels (pg ml^−1^) and tumour PI.

**Figure 4:**
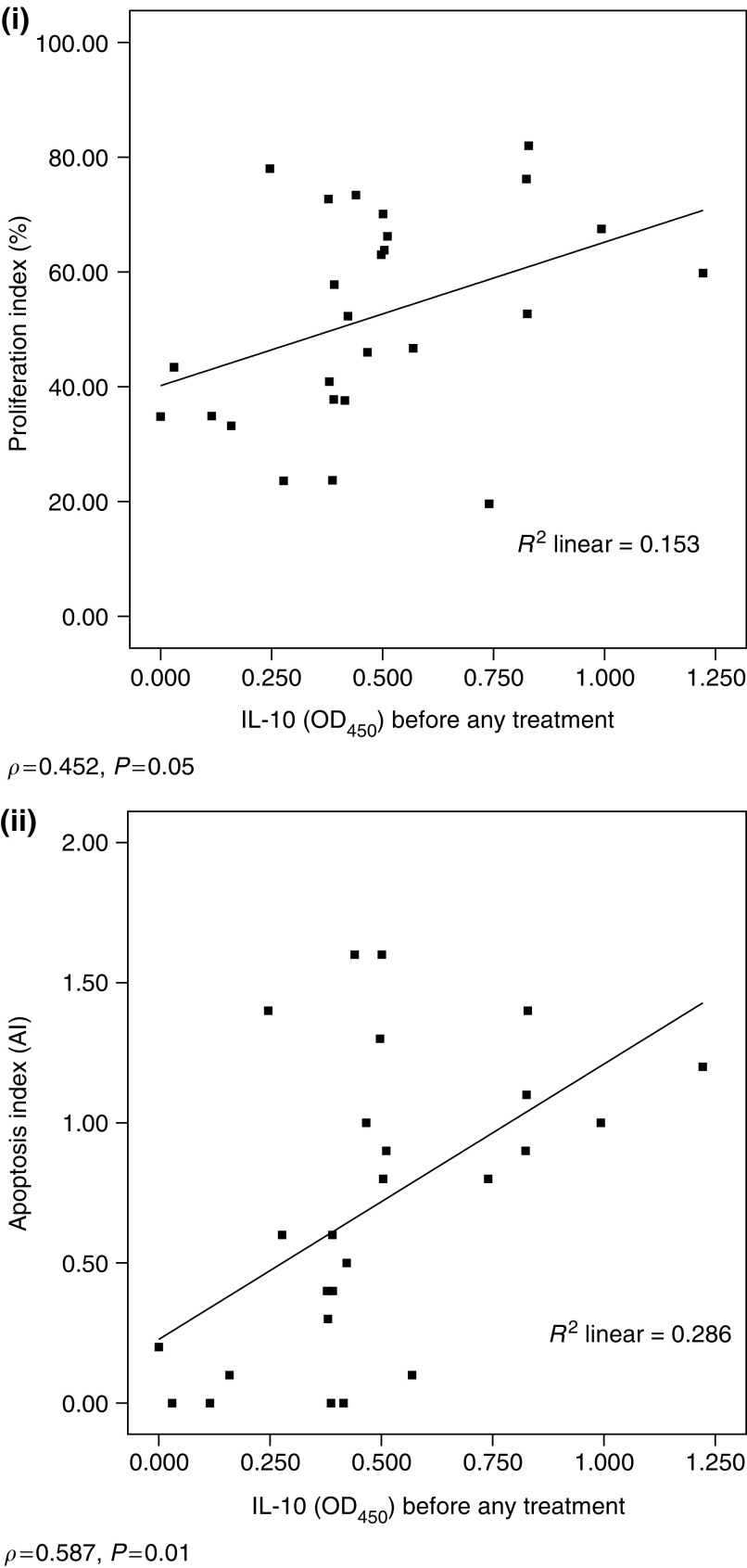
(i) Correlation between preoperative IL-10 levels (OD_450_) and tumour PI. (ii) Correlation between preoperative IL-10 levels (OD_450_) and tumour AI.

A significant negative correlation was found between systemic TNF-*α* levels and tumour cell proliferation (*ρ*=−0.697, *P*=0.01) (Figure 3). A trend to negative correlation was seen when TNF-*α* levels were correlated to apoptosis (*ρ*=−0.33) and IFN-*γ* levels to proliferation (*ρ*=−0.265). Patient systemic IL-10 levels had a positive correlation with both tumour cell proliferation (*ρ*=0.452, *P*=0.05) and apoptosis (*ρ*=0.587, *P*=0.01) (Figure 4(i) and (ii)).

### Correlations to long-term prognosis

**Figure 5:**
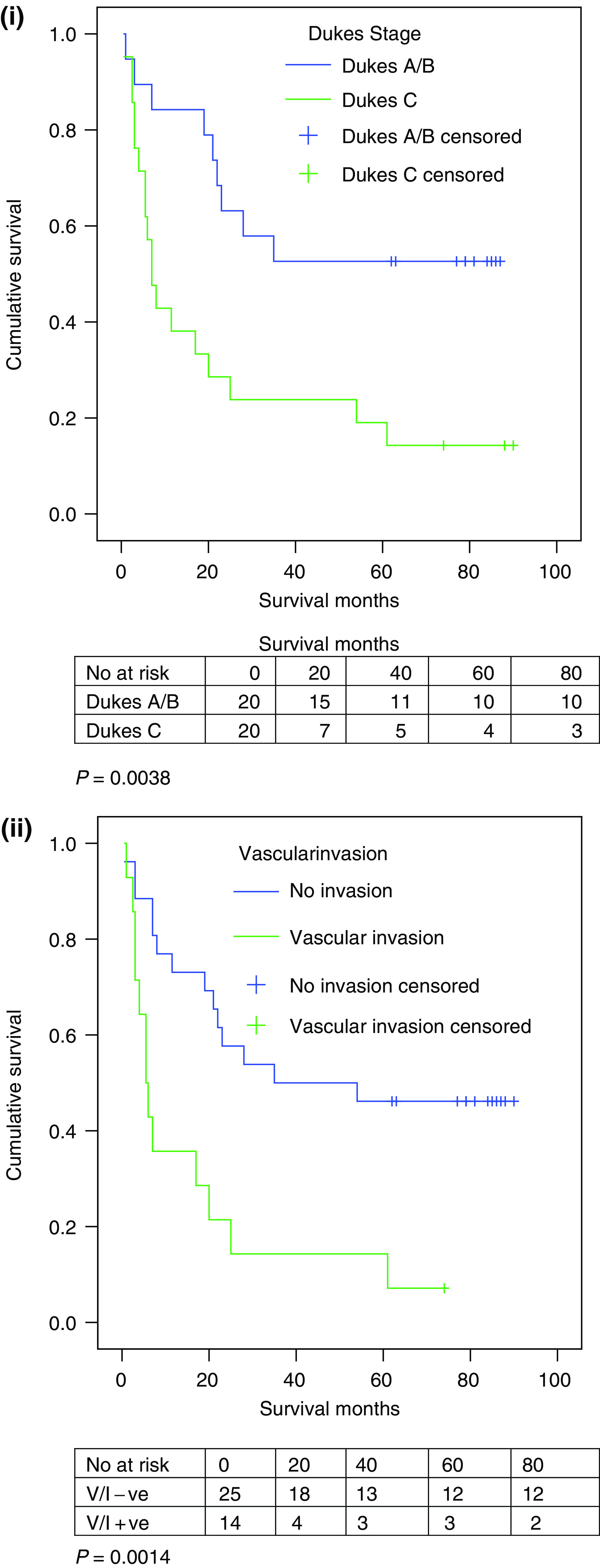
(i) Dukes survival functions. (ii) Vascular invasion survival function.

It was shown that the tumour's pathological staging was of significance when assessing which factors influenced survival. In keeping with the expected survival distribution of patients with colorectal cancer, those with Dukes’ C tumours had a poorer survival compared to those with either a Dukes’ A or B tumour (*P*=0.0038). Vascular invasion was also correlated to reduced survival (*P*=0.0014) (Figure 5(i) and (ii)).

#### Tumour rates of proliferation and apoptosis

**Figure 6:**
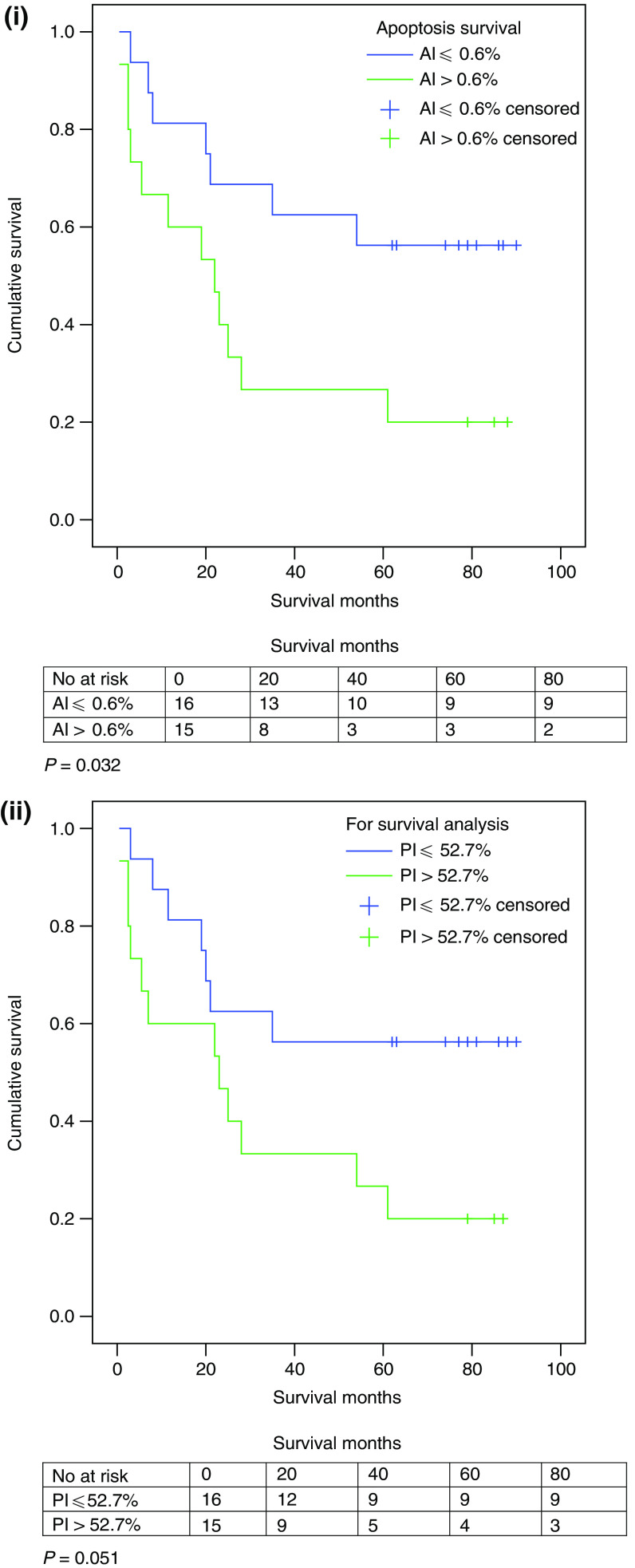
(i) AI survival functions. (ii) PI survival functions.

Patients who had an AI less than or equal to the median value of 0.6% (*n*=16) had significantly better overall survival than those whose value was greater (*n*=15) (log-rank *P*=0.032) (Figure 6(i)). Patients who had a PI less than or equal to the median value of 52.7% (*n*=16) showed a strong trend to improved survival (log-rank *P*=0.051) (Figure 6(ii)).

#### Systemic cytokine levels

Using independent *t*-tests preoperative cytokine levels were compared to see what correlation they had with long-term survival, development of metastases and local recurrence. Patients with a survival of greater than 1 year had significantly higher levels of preoperative TNF-*α* than those that did not (*P*=0.005). There was no correlation between preoperative IFN-*γ* and IL-10 levels and 1-year survival. Preoperative cytokine levels were not significantly different in those who developed metastases or local recurrence compared to those who did not and there were no statistically significant correlations with postoperative cytokine levels.

**Figure 7:**
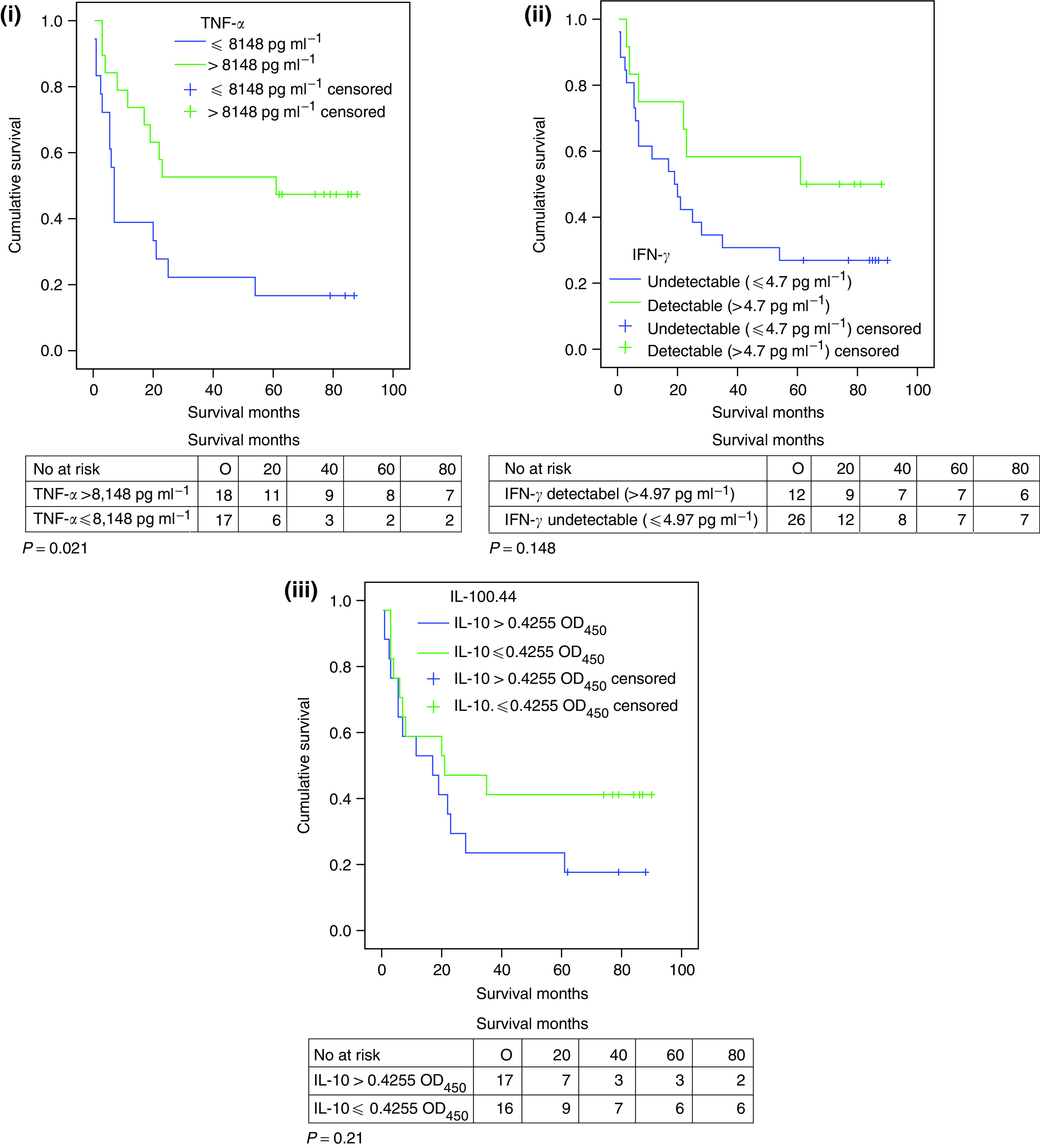
(i) TNF-*α* survival functions. (ii) IFN-*γ* survival functions. (ii) IL-10 survival functions.

In order to perform survival analysis patients were classified in to two groups according to their cytokine values. When assessing the influence of TNF-*α* patients were grouped as either having values ⩽the median value of 8148 pg ml^−1^ (*n*=17) or >8148 pg ml^−1^ (*n*=18). To assess IL-10 patients were split depending whether their values were > or ⩽the median value of 0.4255 OD_450_ (*n*=17 and *n*=16, respectively). However, to assess IFN-*γ* patients were split in to those with values detectable (>4.97 pg ml^−1^) (*n*=12) or not detectable (⩽4.7 pg ml^−1^) (*n*=26) owing to a skewed patient distribution. Patients with TNF-*α* levels >8148 pg ml^−1^ had significantly better survival than those with TNF-*α* ⩽8148 pg ml^−1^ (*P*=0.021) (Figure 7(i)). Patients with detectable IFN-*γ* levels showed a trend to improved survival compared to those with undetectable levels, however, not reaching significance (*P*=0.148) (Figure 7(ii)). There was no statistical difference between the two IL-10 groups although at 60 months it is noted that cumulative survival is more than double in the group with values ⩽0.4255 OD_450_ (0.41 *vs* 0.18) (Figure 7(iii)).

Multivariate analysis performed to assess the significance of independent factors upon 5-year survival demonstrated tumour differentiation, AI and detectable IFN-*γ* levels to be of statistical significance (Table 1).

## DISCUSSION

Colorectal carcinomas arise through a series of histological changes, each of which is accompanied by specific genetic alterations in a handful of oncogenes and tumour-suppressor genes (Fodde *et al*, 2001). Chronic inflammation plays a causative role in the molecular pathways of colon carcinogenesis (Itzkowitz and Yio, 2004) and leads to altered rates of cell proliferation and apoptosis, altered cellular adhesion, increased angiogenesis and cellular transformation (Macarthur *et al*, 2004). Significantly, chronic inflammation also causes negative feedback inhibition of the CMI and causes a predominance of HI. This state of host immunity is described in a number of malignant diseases and inhibits immune-mediated tumour rejection (O’Byrne and Dalgleish, 2001).

Colorectal cancer has been shown to cause CMI suppression and HI promotion that is linked to disease progression and has an influence on survival. Decreased total numbers of the CMI promoting Th1 CD4^+^ cells have been found in patients with colorectal tumours (Nakayama *et al*, 2000). Lowered levels of CMI-associated cytokines have been measured, corresponding to the pathological stage of the tumour and reversed by surgery (O’Hara *et al*, 1998; Heriot *et al*, 2000; Galizia *et al*, 2002; Shibata *et al*, 2002). It has also been demonstrated that elevated basal levels of the HI-associated cytokine, IL-10, are correlated to reduced disease-free survival (Galizia *et al*, 2002). The mechanisms through which this shift in immunity is performed have not been fully explained but include COX-2 overexpression (O’Byrne and Dalgleish, 2001), histamine production (Tomita and Okabe, 2005) and increased expression of IL-10 (Ding *et al*, 2003).

There is evidence of an immune response to colorectal cancer which can be correlated to improved survival (Naito *et al*, 1998); however, many of the cell types active in chronic inflammation can be found in the surrounding stroma and the neoplasm itself. This includes a diverse leucocyte infiltrate of macrophages, neutrophils, eosinophils and mast cells (Macarthur *et al*, 2004). Thus, the secretion of extracellular proteases, proangiogenic factors, and cytokines in the tumour microenvironment results in a chronic inflammatory-like state in which CMI suppression takes place and the control mechanisms for cell proliferation and apoptosis are disturbed.

Despite there being only a small number of patients involved in this study, it is the consistency in its results, relating rates of proliferation and apoptosis to systemic cytokine levels and the trends seen when looking at long-term survival, that suggest progressive changes to host immunity with colorectal cancer advancement. Tumour rates of proliferation and apoptosis have a negative correlation with the CMI-associated cytokines TNF-*α* and IFN-*γ* while having a positive one with IL-10 (HI associated). Elevated AI and PI were linked with reduced long-term survival and the development of metastases and/or local recurrence. Correspondingly, reduced rates of survival are seen with lowered levels of TNF-*α*. It is not possible to comment from these data whether a specific cytokine level or tumour proliferation or apoptosis rate has a prognostic value and it should be noted that a larger range of cytokines and T-cell responses would need to be measured to make a full evaluation of any shift in host immunity from CMI to HI. However, it can be concluded that there is an impact upon patient survival through the systemic suppression of the CMI-associated cytokines TNF-*α* and IFN-*γ* and promotion of the HI-associated IL-10.

The balance between cell proliferation and cell loss through apoptosis determines how fast a tumour grows and is an important determinant of tumour behaviour (Koornstra *et al*, 2003). There are conflicting reports regarding the implications of proliferation and apoptosis rates upon patient prognosis (Koornstra *et al*, 2003; Salminen *et al*, 2005; Valera *et al*, 2005), however, it is generally accepted that as tumours advance proliferative activity increases and higher rates of apoptosis are seen (Koornstra *et al*, 2004). This study's results conform to these findings demonstrating increased rates of proliferation and apoptosis in the group of patients developing either metastatic disease or local recurrence with a strong positive correlation between PI and AI also noted. The demonstrated link between increased rates of apoptosis and proliferation with reduced survival in this cohort of patients could possibly be explained by this pathological-proliferation/apoptosis correlation.

The relationship between cytokines and tumour development is complex with both stimulation and inhibition exhibited by the same one cytokine. Their action depends upon the cocktail of other factors in the surrounding microenvironment, including other cytokines, chemokines, growth and angiogenic factors as well as its own actual concentration. For example, TNF-*α* can induce apoptosis in tumour cells of the colon (Jacobson-Brown and Neuman, 2004) and its cytotoxic effect is synergistically enhanced by IFN-*γ in vitro* and *in vivo* (Sveinbjornsson *et al*, 1997). However, when chronically produced TNF may act as an endogenous tumour promoter, contributing to tissue remodelling and stromal development necessary for tumour growth and spread (Balkwill and Mantovani, 2001). Interleukin-10 is believed to contribute to tumour development by suppressing the antitumour immune responses (Matsuda *et al*, 1994). Gene therapy studies, however, have demonstrated IL-10-mediated inhibition of tumour growth and metastases with the outcome appearing dependent upon host immune competency and levels of IL-10 expression (Erdman *et al*, 2003).

Measuring systemic levels of three cytokines cannot accurately represent the specific interactions occurring in the tumour microenvironment or give a full representation of systemic immunity. However, it does enable potential trends or changes in host immune competence to be proposed. Thus, this study suggests a potential shift in host immunity with CMI suppression and HI promotion, which correlates to increasing tumour rates of proliferation and apoptosis. The effect of any one single cytokine's is not implicated.

These results linking the host's immune response to colorectal cancer progression and prognosis highlight the need for immunotherapies, which enhance CMI. Manipulating the host's systemic immune response to a tumour may lead to an environment in which tumour cell recognition is more successfully induced (Shibata *et al*, 2002) and several studies have demonstrated that CMI-associated cytokines enhance the therapeutic efficacy of antitumour responses (Nakamori *et al*, 2003). Combining immunotherapies with other treatment modalities including chemotherapy and radiotherapy may also lead to more successful results.

## CONCLUSION

There is a trend to TNF-*α* and IFN-*γ* suppression with IL-10 promotion as colorectal cancer advances. Cytokine levels correlate to increased tumour rates of proliferation and apoptosis. Patient survival is influenced by the histological stage of the tumour, vascular invasion, tumour rates of apoptosis and proliferation and systemic immunity. All of these factors are interconnected. Immunotherapies that can enhance cell-mediated immunity may lead to prolonged survival and should be considered in combination with other treatments used to induce tumour recognition.

## Figures and Tables

**Table 1:** Multivariate analysis of significant factors associated with overall 5-year survival in colorectal cancer

	**Regression coefficient**	**s.e.**	**Hazard ratio**	P*
Dukes’ stage (A/B) *vs* (C)	0.687	1.23	1.99 (0.18, 22.00)	0.576
Differentiation (well/mod) *vs* (poor)	−2.065	0.88	0.127 (0.23, 0.71)	0.019
Vascular invasion				
(+ve *vs* −ve)	-2.903	1.62	0.055 (0.01, 1.32)	0.074
Proliferation index (continuous variable)	-0.021	0.04	0.98 (0.91, 1.06)	0.609
Apoptosis index (continuous index)	6.691	3.05	805.12 (2.02, 320256.16)	0.028
TNF-*α* (>8148 pg ml^−1^ *vs* ⩽8148 pg ml^−1^)	-0.983	1.42	0.374 (0.023, 6.05)	0.489
IFN-*γ* (>10 pg ml^−1^ *vs* ⩽10 pg ml^−1^)	5.211	2.22	183.33 (2.35, 1433.71)	0.019
IL10 (> 0.4255 OD_450_ *vs* ⩽0.4255 OD_450_)	1.640	1.47	5.153 (0.29, 91.24)	0.263

Values in parentheses are 95% CI. ^*^Cox proportional hazards model.
